# Assessment of a Rabies Virus Rapid Diagnostic Test for the Detection of Australian Bat Lyssavirus

**DOI:** 10.3390/tropicalmed3040109

**Published:** 2018-10-04

**Authors:** Andrea Certoma, Ross A. Lunt, Wilna Vosloo, Ina Smith, Axel Colling, David T. Williams, Thao Tran, Stuart D. Blacksell

**Affiliations:** 1 CSIRO Australian Animal Health Laboratory, Portarlington Rd, East Geelong, VIC 3218, Australia, andrea.certoma@csiro.au (A.C.); Ross.Lunt@csiro.au (R.A.L.); Wilna.Vosloo@csiro.au (W.V.); Ina.Smith@csiro.au (I.S.); axel.colling@csiro.au (A.C.); D.Williams@csiro.au (D.T.W.); 2 Mahidol-Oxford Tropical Medicine Research Unit, Faculty of Tropical Medicine, Mahidol University, Bangkok 10400, Thailand; trantxthao@gmail.com; 3 Centre for Tropical Medicine, Nuffield Department of Clinical Medicine, Churchill Hospital, Oxford OX3 7FZ, UK

**Keywords:** Australian bat lyssavirus, rabies Ag detection rapid test, fluorescent antibody test

## Abstract

Australian bat lyssavirus (ABLV) is closely related to the classical rabies virus and has been associated with three human fatalities and two equine fatalities in Australia. ABLV infection in humans causes encephalomyelitis, resulting in fatal disease, but has no effective therapy. The virus is maintained in enzootic circulation within fruit bats (*Pteropid* spp.) and at least one insectivorous bat variety (*Saccolaimus flaviventris*). Most frequently, laboratory testing is conducted on pteropodid bat brains, either following a potential human exposure through bites, scratches and other direct contacts with bats, or as opportunistic assessment of sick or dead bats. The level of medical intervention and post-exposure prophylaxis is largely determined on laboratory testing for antigen/virus as the demonstrable infection status of the in-contact bat. This study evaluates the comparative diagnostic performance of a lateral flow test, Anigen Rabies Ag detection rapid test (RDT), in pteropodid variant of ABLV-infected bat brain tissues. The RDT demonstrated 100% agreement with the reference standard fluorescent antibody test on 43 clinical samples suggesting a potential application in rapid diagnosis of pteropodid variant of ABLV infection. A weighted Kappa value of 0.95 confirmed a high level of agreement between both tests.

## 1. Introduction

*Australian bat lyssavirus* (ABLV) is a species of the *Lyssavirus* genus in the *Rhabdoviridae* family, and is closely related to classical rabies virus [1]. ABLV was first identified in 1996 and has been associated with three reported human fatalities, two in adults and another in a child [2,3,4]. Naturally occurring equine infection has also been reported [5]. ABLV circulates in frugivorous bats of the *Pteropus* species and insectivorous bat species, *Saccolaimus flaviventris* [6], and there is serological evidence of infection in bats belonging to genera of the *Megadermatidae*, *Hipposideridae*, *Mollosidae* and *Vespertilionidae* families [7]. Prevalence of ABLV infection in sick, injured or orphaned bats has been reported as 6–9% [8,9]. Closely-related viruses may circulate more widely as evidenced by serological detection of ABLV neutralizing antibodies in bats in the Philippines [10]. Clinically, ABLV-associated disease is similar to rabies, affecting mammals and causing an acute viral encephalomyelitis. ABLV infection in humans is rare but fatal, and without effective treatment. However, ABLV infection may be prevented by administration of a post-exposure prophylaxis regimen of human rabies immunoglobulin and rabies vaccine [11].

Australia is considered to be free of the rabies virus despite the close relationship between the rabies virus and ABLV. The emergence of ABLV has proved to be a diagnostic challenge, as potentially affected bats may be located remotely across all mainland States and Territories, while confirmation of ABLV-infected cases is only performed at specialized laboratories, including the Australian Animal Health Laboratory (AAHL) in Geelong. The gold standard for detection of ABLV is the fluorescent antibody test (FAT) of brain tissue, which requires specialized skills and a fluorescence microscope [12]. Other diagnostic tests for detection of lyssavirus, including the tissue culture infection test, mouse inoculation test, direct rapid immunohistochemistry test and RT-PCR, are laboratory-based tests that require specialized equipment and reagents [12,13,14,15]. Availability of a reliable rapid test for diagnosis of ABLV in Australia could expedite patient at-risk assessment determinations, or management of domestic animals that have had contact exposure to a potentially infected bat, by testing the infection status of the bat involved in the exposure.

A lateral flow test using immunochromatographic principles, called Anigen Rabies Ag detection rapid test (RDT) (Bionote, Animal Genetics, Inc., Gyeonggi-Do, Korea), is commercially available for detection of rabies virus antigen in brain and saliva samples. It has previously been demonstrated that the RDT can detect all representative rabies genotypes in Africa when compared to an indirect FAT (IFAT) [16]. Limited testing of other lyssavirus species, including European bat lyssavirus (EBLV) types 1 and 2, Bokeloh bat lyssavirus (BBLV) and Duvenhage virus (DUVV), showed that only BBLV was detected by RDTs [17]. The aim of the study presented here was to undertake the first assessment of an RDT for diagnosis of ABLV infection in fruit bat brain homogenates. The RDT demonstrated highly comparable diagnostic performance to the reference standard FAT in detection of ABLV in a limited number of well-characterized brain tissues sampled from Australian bats.

## 2. Materials and Methods

The archived bat brain tissues (43 samples) used in this study were collected from 1997 to 2009 as diagnostic submissions to the Australian Animal Health Laboratory (AAHL) for lyssavirus detection by FAT using an FITC-conjugated monoclonal antibody (Fujirebio-Europe, Ghent, Belgium) [18]. The samples included 24 FAT lyssavirus-positive brain tissue specimens from *Pteropus* spp. and 19 FAT lyssavirus-negative brain tissue from a range of bat species. Confirmatory testing was also undertaken for 20 of the FAT-positive samples using conventional or real-time RT-PCR assays specific for pteropid ABLV [19,20], with 17 samples confirmed as positive. The brain tissue samples generally represented a pool of brain stem, medulla, hippocampus and cortex regions. A 10–20% (*w*/*v*) homogenate of each brain was prepared in phosphate buffered saline (pH 7.3) and held at −80 °C following initial diagnostic testing. All 43 samples were tested with the RDT, using the method outlined by Kang et al. [21]. The RDT uses gold-conjugated detector antibodies combined with a monoclonal antibody directed against the lyssavirus nucleocapsid protein that is adsorbed to the ‘test line zone’ of the device that indicates the sample result and a goat anti-mouse IgG is adsorbed to the ‘control line zone’ that indicates the validity of the RDT result [21]. Each sample was thawed to ambient temperature and diluted with an equal volume of the diluent provided in the kit. The suspension was incubated at room temperature for 10 min. Four drops (or approximately 125 µL) were loaded into the device, using the transfer pipette supplied with the kit, and incubated for 5 min at room temperature. The test results were read within 5–10 min after incubation, as specified by the manufacturer. All laboratory testing was conducted within biosafety level 3 facilities at the AAHL by rabies-vaccinated personnel.

Groups of pteropodid variant of ABLV positive (*n* = 24) and negative (*n* = 19) samples were used to establish preliminary performance characteristics between the reference standard (FAT) and candidate test (RDT). Results for sensitivity (Se) and specificity (Sp) were expressed as point estimates with 95% confidence intervals and in a 2 × 2 table [22,23]. To evaluate the level of agreement between tests, Kappa agreement was calculated using point estimates and 95% CI. To avoid overly optimistic assessments, a weighted version of Kappa with negative, weak positive and positive results was applied [24]. Data analysis was performed with MedCalc statistical software package bvba (MedCalc software, Ostend, Belgium).

## 3. Results

This study evaluated the performance of the Anigen RDT in the diagnosis of pteropodid variant of ABLV infection in pteropodid bat brain tissues compared to the reference standard FAT. There was 100% agreement between the RDT and FAT results for all positive and negative brains tested (Table 1 and Table 2). The RDT demonstrated an absence of false-positivity in all 19 FAT-negative samples that were derived from various bat species, resulting in 100% specificity (95% CI (0.82, 1.00)). All 24 lyssavirus-positive brains originally detected by FAT were positive using the RDT, demonstrating 100% sensitivity (95% CI (0.86, 1.00)) for the latter test. It should be noted that the level of reactivity observed with the RDT varied between samples; for example, sample 02-02283 was only weakly positive in the RDT but a clear positive in the FAT (Table 1). This suggested potential loss of sensitivity from sample degradation following freeze-thaw or that the RDT has slightly less analytical sensitivity than the FAT.

Using the same set of data, but with one weak positive result in the RDT, a weighted Kappa analysis was performed to estimate the level of agreement between tests beyond chance, and resulted in a point estimate of 0.95 (95% CI [0.87–1]). Kappa values between 0.81 and 1 are considered to be very good and indicate a high level of agreement.

## 4. Discussion

The results presented here demonstrated high levels of relative sensitivity and specificity for the rabies RDT with pteropodid variant of ABLV positive and negative pteropodid bat samples. Previously published reports of diagnostic sensitivity estimates for the rabies RDT with RABV ranged from 91.7% to 96.9% and were associated with high specificity (98.9% to 100%) when compared to the FAT [25]. However, the recent study of Eggerbauer et al. [17] reported that RABV RDTs from multiple manufacturers, including Anigen, were not as sensitive as previously found for the detection of RABV. This study also showed that the Anigen RDT was able to detect BBLV, but not DUVV or EBLV in infected brains samples [17]. Our study provides the first evidence that this test is also suitable for the detection of pteropodid variant of ABLV. In this regard, the results reported here also highlight the utility of the lyssavirus nucleocapsid protein, which is used as the RDT diagnostic target. The nucleocapsid protein is conserved among this virus species; indeed, the nucleocapsid protein of ABLV shares 92% identity with that of the Pasteur vaccine rabies strain [26].

A significant limitation of this study was that all of the ABLV-positive brains tested were from *Pteropus* spp. fruit bats, which were the most common samples submitted to the AAHL. It would have been optimal to test samples from a wider range of hosts, including from *Saccolaimus* species of microbats, which have been shown to be infected with a different strain of ABLV to that of pteropid bats [27]. Other relevant animal species for the laboratory diagnosis of ABLV infection include horses [5] and companion animals such as dogs and cats that have had contact with bats, and for which diagnostic testing is occasionally performed at AAHL. However, only two equine cases have been reported [5] and there is only experimental evidence of infection of dogs and cats [28]. Specimens from these animals were not available for testing in this study. In other studies, the RDT was reported to detect rabies antigen in brain specimens from several species of animals, including dogs, cats, racoons, mongoose, jackals, civet, hyenas, camels and buffalos [17,29]. Further studies are required to determine if ABLV-positive samples from non-bat species will also be detected using the Anigen rabies RDT.

Availability of an accurate rapid test, such as the RDT, may be beneficial as a low-cost alternative to the FAT or PCR tests, which require specialized equipment and a high level of staff training. The RDT kit may be stored at room temperature and is simple to use. It also has potential application in remote/field laboratory settings with appropriate biosafety measures, where it may be a useful tool for preliminary diagnosis of suspected lyssavirus infected bats and follow-up confirmation of samples with established and validated laboratory tests. However, further assessment and validation of this test is required to prove its utility especially with representative species and rabies strains. In particular, testing of samples from animals infected early (analytical Se) and repeatability/reproducibility assessments will be necessary to prove its fitness for purpose.

## Figures and Tables

**Table 1:** Comparison of the Anigen Rapid Rabies Ag Test Kit (RDT) with the reference standard fluorescent antibody test (FAT) using clinical specimens derived from brain tissues of Australian pteropodid bats.

Bat Species	SAN	FAT FAT RESULT	RDT KIT RESULT
*Pteropus poliocephalus* (PC)	04-02171	+	+
*Pteropus poliocephalus* (GHFF)	09-03883	+	+
*Pteropus poliocephalus*	09-01140	+	+
*Pteropid alecto* (black flying fox)	07-01227	+	+
*Pteropid poliocephalus*	07-00872	+	+
*Pteropus alecto*	07-00344	+	+
*Pteropus alecto*	07-00242	+	+
*Pteropus poliocephalus*	04-03133	+	+
*Pteropus poliocephalus*	04-02178	+	+
*Pteropus poliocephalus*	05-08494	+	+
*Pteropus poliocephalus*	05-08728	+	+
*Pteropus poliocephalus*	06-04188	+	+
*Pteropus alecto*	05-05791	+	+
*Pteropus alecto*	05-00252	+	+
*Pteropus* sp.	02-02266	+	+
*Pteropus scapulatus*	02-02283	+	+ weak
*Pteropus poliocephalus*	02-01225	+	+
*Pteropus poliocephalus*	00-00573	+	+
*Pteropus* sp.	01-02752	+	+
*Pteropus poliocephalus*	01-00593	+	+
*Pteropus poliocephalus*	97-01679	+	+
*Pteropus poliocephalus*	97-01680	+	+
*Pteropus alecto*	97-01678	+	+
*Pteropus scapulatus*	97-01677	+	+
*Pteropus scapulatus* (NC)	09-01659	−	−
*Miniopterus schreibersii* (large bent winged bat)	09-00147	−	−
*Chalinolobus morio* (chocolate wattled bat)	09-00260	−	−
*Pteropus poliocephalus*	09-00268	−	−
Microbat (unidentified)	09-00280	−	−
*Pteropus scapulatus*	09-00546	−	−
Microbat (unidentified)	09-00571-1	−	−
Microbat (unidentified)	09-00630	−	−
*Pteropus poliocephalus*	09-00982	−	−
Microbat (unidentified)	09-01370	−	−
*Nyctophilus geoffroyi*	09-01761	−	−
*Pteropus* sp.	09-02604	−	−
*Pteropus poliocephalus*	09-02459	−	−
Long eared bat	09-01830	−	−
*Nyctophilus geoffroyi* (lesser long eared bat)	09-01761	−	−
*Pteropus poliocephalus*	09-02934	−	−
*Pteropus* sp.	09-03271	−	−
*Chalinolobus gouldii*	09-03449	−	−
*Pteropus poliocephalus*	09-04309	−	−

+ denotes positive test result, − denotes negative test result. SAN: sample admission number, FAT: fluorescent antibody test, RDT: Anigen Rabies Ag detection rapid test, PC: positive control from infected bat brain, NC: negative control from non-infected bat brain, GHFF: grey headed flying fox.

**Table 2:** Summary of results shown in Table 1 in a 2 × 2 table, for calculation of relative Sensitivity and Specificity of the rapid diagnostic test (RDT) using the fluorescent antibody test (FAT) as reference test.

RDT Result		FAT		
		Positive	Weak Positive	Negative
	Positive	23	0	0
	Weak positive	1	0	0
	Negative	0	0	19
	Total	24	0	19
